# An updated checklist of Scyphozoa (Cnidaria, Medusozoa) from the Mexican Caribbean: integrating literature, citizen science and field collections

**DOI:** 10.3897/BDJ.14.e182574

**Published:** 2026-01-27

**Authors:** Edgar Gamero-Mora, Ivan A. Castellanos Osorio, Laura Celis, María A. Mendoza-Becerril

**Affiliations:** 1 Medusozoa México, La Paz, Mexico Medusozoa México La Paz Mexico; 2 El Colegio de la Frontera Sur (ECOSUR), Chetumal, Mexico El Colegio de la Frontera Sur (ECOSUR) Chetumal Mexico

**Keywords:** jellyfish, Mesoamerican Barrier Reef, non-native species, taxonomy

## Abstract

**Background:**

This study presents an updated checklist of Scyphozoa from the Mexican Caribbean, incorporating records from published literature, citizen-science initiatives and collected specimens.

**New information:**

This study documents evidence for the presence of 17 scyphozoan taxa, including nine new records for the region. Notably, the checklist includes records of the non-native scyphozoan species *Cassiopea andromeda* and *Mastigias* sp., reflecting the advantage of combining multiple sources of information. Most studies on scyphozoan ecology, distribution, abundance and diversity in the Mexican Caribbean have focused on shallow costal environments (< 30 m), leaving deep-sea and oceanic zones understudied. This uneven sampling effort, together with the difficulties in identifying certain species, hinders our complete understanding of jellyfish biodiversity in the region. Future studies integrating morphological and molecular approaches are essential to resolve taxonomic uncertainties and fully characterise scyphozoan biodiversity in this region.

## Introduction

Cnidaria comprises three major clades: Anthozoa, Endocnidozoa and Medusozoa (Ohdera et al. 2019). Medusozoa is remarkable amongst cnidarians due to the medusa being a key stage in its life cycle (Collins 2002). Although a single origin of the medusa is proposed, this stage has been repeatedly lost or reduced within the group throughout evolution (Collins 2002, Ames 2018), a pattern that is significantly associated with differences in diversification rates amongst clades (Morales-Guerrero 2021) and, consequently, with uneven species richness across them. Scyphozoa is a clade within Medusozoa with a high number of species. Its species richness is, therefore, relevant for studies of regional marine biodiversity.

Scyphozoa, commonly known as true jellyfish, are classified into two subclasses, Coronamedusae and Discomedusae and three extant orders: Coronatae, Rhizostomeae and Semaeostomeae. Scyphozoans typically exhibit complex life cycles with a planula larva, benthic polyp stage and pelagic medusa phase (Collins 2002, Collins et al. 2006). Polyps can reproduce asexually by budding to produce clones of themselves or through a process called strobilation to generate young jellyfish - ephyrae - that mature into adults (Arai 1997, Sukhoputova et al. 2018). As they live through distinct life stages, they can thrive in varied environments while also playing a key role in ocean food webs (Purcell 1985, Stoltenberg et al. 2021) alongside Earth’s chemical cycles (Hubot et al. 2022).

True jellyfish play a critical role in marine ecosystems mainly due to their dominant medusa stage, high feeding rates and their capacity to rapidly increase in abundance under favourable conditions (Macalia and Bergami 2020). By feeding on zooplankton, fish eggs and larvae, as well as juvenile fish, jellyfish influence the structure of planktonic communities (Gibbons et al. 2015). Their voracity and high reproductive rates allow them to temporarily dominate certain marine areas, giving rise to jellyfish blooms (Purcell 2005, Gibbons et al. 2015). In addition, the accumulation of jellyfish in coastal industrial facilities can block refrigeration systems and result in operational interruptions and additional costs (Kennerley et al. 2022). However, these blooms can be exploited, for example, by the pharmaceutical industry, since, due to the particular characteristics of jellyfish of this class, it is believed that collagen derived from them could generate innovative and attractive commercial products of interest to humans, with distinctive functional and physicochemical properties (Chiarelli et al. 2023).

Globally, the group comprises about 244 species (Collins et al. 2025) and occurs throughout the world’s oceans (Jarms and Morandini 2023, OBIS 2025). The main inventories of true jellyfish that include Mexican waters have focused on the medusa stage, reporting 16 species for the coast of Mexico (Segura-Puertas et al. 2003), 19 species for Gulf of Mexico (Segura-Puertas et al. 2009), 20 species for the coast of Mexico (Gasca and Loman-Ramos 2014) and 35 species for Mexican Pacific (Estrada-González et al. 2023). The Mexican Atlantic coast comprises two contrasting, but oceanographically connected regions (Murphy et al. 1999). The Gulf of Mexico, a semi-enclosed basin with turbid waters and a high sediment load from rivers and coastal lagoons (De la Lanza Espino et al. 2013) and the Mexican Caribbean, of karstic origin and dominated by the Yucatan Current, have oligotrophic, transparent and stable ocean waters that favour the development of reef ecosystems (Merino-Ibarra and Otero-Dávalos 1991).

Despite this connectivity, in the Mexican Caribbean, the species richness of this group is inconsistent amongst sources. While Segura-Puertas et al. (2003) and Gasca and Loman-Ramos (2014) both reported eight species, global inventories recognise 15 species for the region (Jarms and Morandini 2019) or only two, according to the Ocean Biodiversity Information System (OBIS 2025). These discrepancies likely reflect differences in taxonomic criteria, sampling effort, geographic scope and data sources. While primarily literature-based, some inventories incorporate novel observations from ROVs (Remotely Operated Vehicles) and other technologies. As biodiversity assessments are increasingly moving beyond traditional literature-based inventories towards more integrative approaches, alternative data sources are gaining relevance, with citizen science representing an important tool for biodiversity assessments in Mexican waters (Puente-Tapia et al. 2021, Villalobos-León and Mendoza-Becerril 2025).

Other jellyfish research in the Mexican Caribbean Sea has documented species composition, community structure and spatial or temporal variation across coastal and offshore environments (Segura-Puertas 1992, Segura-Puertas and Ordóñez-López 1994, Segura-Puertas and Damas-Romero 1997, Suarez-Morales et al. 1999, Suárez-Morales et al. 1999, Gasca et al. 2003, Canché-Canché and Castellanos-Osorio 2005). Additional studies on true jellyfish in the region have focused on the toxicological properties of *Aurelia aurita* (Linnaeus, 1758), *Cassiopea xamachana* Bigelow, 1892, *Pelagia noctiluca* (Forsskål, 1775) and *Linuche unguiculata* (Swartz, 1788), addressing haemolytic, electrophysiological, antitumoral and molecular effects of their venoms (Torres et al. 2001, Orduña-Novoa et al. 2003, Ponce et al. 2013, Morales-Landa et al. 2021). These studies not only provide important information on the biology and ecological roles of scyphozoans in the region, but also represent valuable sources of species records that contribute to understanding their richness; however, the knowledge available since 2014 remains scattered across isolated studies (Gasca and Loman-Ramos 2014), underscoring the need for updated and integrated inventories.

Despite their ecological relevance, the biodiversity of these jellyfish in the Mexican Caribbean remains insufficiently documented, largely because available information is scattered across isolated studies and heterogeneous data sources rather than integrated into a single regional synthesis. Therefore, there is a need to promote scientific efforts to help address the fragmentation of existing information and provide a reference framework for future taxonomic, ecological and biogeographic studies. In this study, we present an updated checklist of Scyphozoa from the Mexican Caribbean, integrating records from published literature, citizen science and collected specimens. Considering that the Mexican Caribbean is part of the Mesoamerican Barrier Reef System — known as one of the most important marine regions worldwide for its high biodiversity and ecological value (Guimarais et al. 2021) — this taxonomic inventory provides a current synthesis and reference point for future studies.

## Materials and methods

### Literature search

Records of scyphozoan jellyfish inhabiting the Mexican Caribbean were gathered from sources published up to May 2025, including peer-reviewed articles, species checklists, theses, book chapters and reports from institutions. When explicit geographic coordinates were not provided in original documents, we applied two strategies: (1) reusing previously published coordinates for the same locality or (2) georeferencing in QGIS 3.34.15 using QuickMapServices and OSM Place Search plugins when maps with sampling sites were available. Georeferencing precision varied from < 100m (identifiable landmarks) to ~ 1-5km (general locality descriptions). Information on the origin of geographic coordinates is provided in the supplementary material deposited in Zenodo (Mendoza-Becerril et al. 2025).

In several cases, the original sources described sampling across many sites or seasons, but did not specify where each species was found. For these instances, a precautionary approach was taken: coordinates were assigned only to a single locality where the species had been reliably recorded in literature, typically reusing coordinates from previous studies when the same site had been documented. Likewise, when articles provided environmental data, but did not specify values per sampling station, the values reported here represent either the range (maximum and minimum) or the average of surface values, as stated by the original authors. Specific notes regarding assumptions, limitations or rationale behind each record (including georeferencing choices and interpretation of hydrological parameters) are presented in Mendoza-Becerril et al. (2025).

### Citizen-science data

Citizen-science records included data from iNaturalist (https://www.inaturalist.org/), personal communication and the Blackwater Cozumel Scuba group. All records were reviewed by the authors to confirm their assignment to Scyphozoa and to assess taxonomic consistency, typically at genus level when species-level identification was not supported by available photographic evidence, as well as the plausibility of their reported geographic location. Search parameters on the website were set to show records of Scyphozoa from the Mexican Caribbean (state of Quintana Roo, Mexico) from the first available data until March 2025 (https://www.inaturalist.org/taxa/48332-Scyphozoa). Records classified as “Research Grade” and “Needs identification” were included. Observations in the “Needs identification” category were carefully reviewed and photo-based verification was used to assess identification. For certain taxa (detailed in the taxonomic remarks), species-level identification was not possible since distinguishing species within these groups requires genetic data due to high morphological similarity. When the coordinates of an iNaturalist observation were located on land, the point was manually corrected by relocating it to the nearest marine area based on geographic context. Records were excluded if they lacked photographs, were taken in aquariums or had coordinates situated far inland with no reasonable correction possible.

In addition, some records were obtained through personal communication and occasional encounters with scyphozoans reported by the Blackwater Cozumel Scuba Diving Centre until May 2025. These records were also checked individually to confirm species identification from photographs and to verify geographic coordinates.

### Samples collected

Scyphozoans were collected and identified along the Mexican Caribbean coast using different sampling methods summarised in Table 1.

Most specimens were preserved in 4% formalin and, therefore, could not be used for molecular analyses. Only five recently-collected specimens of *C. xamachana* were preserved in 96% ethanol (Table 1). For this reason, molecular sequencing was conducted exclusively for this species to verify the taxonomic identity of recently-collected material in a group with known morphological overlap, consistent with previous molecular identifications reported by Gamero-Mora (2014). DNA was extracted from bell tissue samples, which were digested overnight at 56°C using Proteinase K in invertebrate lysis buffer and subsequently purified using a membrane-based glass fibre approach (Ivanova et al. 2006, Elías-Gutiérrez et al. 2008). The isolated DNA was used as a template to amplify a partial region of the mitochondrial 16S gene (~ 610 bp) using the C&B1(F) and C&B2(R) primers (Cunningham and Buss 1993), following the PCR conditions described by Gamero-Mora et al. (2022). PCR products were sequenced using Sanger sequencing and the resulting chromatograms were visually inspected and low-quality regions were trimmed. The sequences were deposited in the Barcode of Life Data Systems (BOLD) and are available under accession code MEDUS (Table 2).

### Species richness estimation and mapping

Records identified only to genus level were included in species richness estimates when they likely represent unique, unresolved taxonomic entities. In contrast, genus-level records interpreted as unassignable observations of already documented species were excluded from species counts. In this regard, the designation “sp.” was applied when records likely correspond to a unique unresolved species, whereas “spp.” was used when observations could represent multiple species already documented for the Mexican Caribbean.

Maps of species distribution in the Mexican Caribbean were generated using SimpleMappr (https://www.simplemappr.net) by plotting records from collected specimens, literature and citizen-science sources. To delimit the study area, we overlapped the Caribbean Region polygon from Wilkinson et al. (2009) on to our maps and no specific coordinates were taken from this source, as the polygon was used only as a geographic reference. Accordingly, the study area included all true jellyfish records falling within this polygon, encompassing both coastal and offshore observations, without restriction to territorial seas or specific depth limits. Final editing and figure adjustments were carried out in Inkscape, available at https://inkscape.org/.

## Checklists

### Scyphozoa Goette, 1887

#### Coronamedusae

Calder, 2009

D81B630C-0D25-5059-9594-9E1DE20E7E1D

#### Coronatae

Vanhöffen, 1892

60CB2621-2952-52F7-8CE7-B3270A01F7F8

#### Atollidae

Hickson, 1906

BB200AC4-C3C2-5194-84A7-00A1D744A592

#### Atolla vanhoeffeni

Russell, 1957

52BB7FED-6228-5FA8-A70C-2BD24C6DE0AF

https://www.marinespecies.org/aphia.php?p=taxdetails&id=135281

##### Distribution

**Figure 1.**
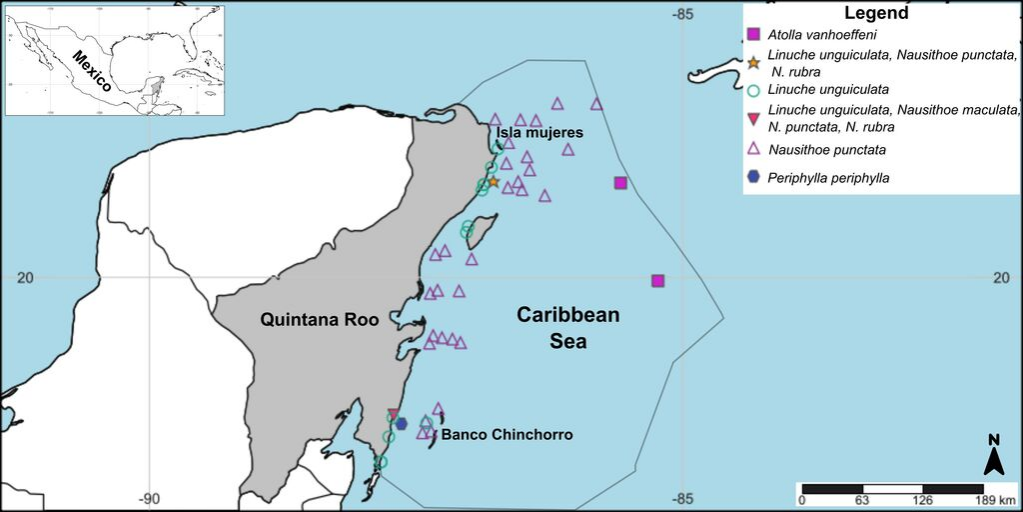
Literature, citizen science and samples collected records of Scyphozoa, Coronate in the Mexican Caribbean from 1972 to 2025. The record of *Nausithoe* sp. is not shown in the map because it is based on a citizen-science observation reported with a broad coordinate range that could not be plotted as a discrete locality.

See Fig. 1, Mendoza-Becerril et al. (2025).

##### Notes

This species belongs to the family Atollidae, a monogeneric family containing 12 valid *Atolla* species (Collins et al. 2025). However, several species remain taxonomically uncertain; only *A. vanhoeffeni*, *A. chuni* Vanhöffen, 1902 and *A. gigantea* Maas, 1897 are well documented. *Atolla vanhoeffeni* can be readily recognised by the presence of eight pigment spots on the subumbrellar wall near the gastric cavity (Matsumoto et al. 2022); notably, *A. vanhoeffeni* is the only species in the genus with a semi-cosmopolitan distribution (Collins et al. 2025). In contrast, *A. chuni* and *A. gigantea* are only mentioned in relation to their type localities with no further distributional data available. While the semi-cosmopolitan distribution of *A. vanhoeffeni* makes its presence in the Caribbean plausible, recent integrative studies have revealed cryptic species within *Atolla* (Matsumoto et al. 2022), raising questions about the reliability of morphology-based identifications. Without voucher material or detailed morphological description from Phillips (1972), this record should be treated as tentative (Atolla cf. vanhoeffeni) pending confirmation Matsumoto et al. (2022).

#### Linuchidae

Haeckel, 1880

E6C9F8A8-94FE-50F5-BDDE-47E9A9C9E50B

#### Linuche unguiculata

(Swartz, 1788)

4EF3602B-17F0-5161-AEE6-671D15D71F3A

https://www.marinespecies.org/aphia.php?p=taxdetails&id=221098

##### Distribution

See Fig. 1, Mendoza-Becerril et al. (2025).

##### Notes

No voucher specimens from the Mexican Caribbean were available for direct examination in this study. However, *Linuche unguiculata* is included here, based on previously published records in the region from literature sources and citizen-science observations. The genus comprises the valid species *L. aquila* (Haeckel, 1880), *L. draco* (Haeckel, 1880) and *L. unguiculata* (Collins et al. 2025). *Linuche aquila* was originally described off eastern Madagascar (Haeckel 1880) and is considered to occur mainly in the Pacific and Indian Oceans (Mayer 1910). Recent observations summarised by Chuan et al. (2021) confirm the presence of *L. aquila* in the Indo-Pacific Region including Borneo and the Philippines. As summarised by Kramp (1961), some authors suggested that *L. draco* is probably a synonym of *L. unguiculata*; however, *L. draco* was recently recorded in Japanese waters (Toshino 2020) supporting its current recognition as a valid species. Based on current knowledge about the distribution of these three species, the most probable identification for specimens in the Mexican Caribbean is *L. unguiculata*, whose type locality is in the Jamaican part of the Caribbean Sea (Collins et al. 2025) (Fig. 2a).

#### Nausithoidae

Haeckel, 1880

59CFDDE8-6B09-5007-B5CF-B28B78E2C826

#### Nausithoe maculata

Jarms, 1990

DF5CD9A6-5B55-552E-8D59-C3B2A9FFB0B4

https://www.marinespecies.org/aphia.php?p=taxdetails&id=287158

##### Distribution

See Fig. 1, Mendoza-Becerril et al. (2025).

##### Notes

The species reported from the Mexican Caribbean was originally identified as *Nausithoe aurea* Silveira & Morandini, 1997 by Tovar Juárez (2003); however, that taxon was later synonymised with *N. maculata* after detailed morphological comparisons (Molinari et al. 2020) and the updated name already appears in the taxonomic list presented by Tovar-Juárez et al. (2025). The type locality of *N. maculata* is Puerto Rico, whereas that of *N. aurea* is Ilhabela, Brazil and this suggests that both names have referred to populations from the western Atlantic possibly connected by large-scale ocean currents. In their revision, Molinari et al. (2023) examined a wide range of specimens assigned to *N. maculata*, including material from Puerto Rico, Belize, Cuba, Brazil and Florida (USA). Most of these specimens lacked depth information and included both wild medusae and individuals reared from polyps collected in shallow water environments. To date, to the best of our knowledge, polyps of *N. maculata* have not been documented from the Mexican Caribbean, though suitable shallow-water substrates exist and targeted surveys have not been conducted. Taken together, this broad western Atlantic distribution (including multiple sites within the Caribbean) supports the validity of the record reported from the Mexican Caribbean by Tovar-Juárez et al. (2025).

#### Nausithoe punctata

Kölliker, 1853

63341F15-1970-59A8-A3B6-1088B4B4A427

https://www.marinespecies.org/aphia.php?p=taxdetails&id=135290

##### Distribution

See Fig. 1, Mendoza-Becerril et al. (2025).

##### Notes

*Nausithoe punctata* was the most frequently recorded coronate medusa in the Mexican Caribbean (Table 2, Fig. 2b). This is a shallow-water species and has a broad distribution, being present in the Mediterranean Sea, Red Sea, North Atlantic and multiple localities in the Caribbean — Puerto Rico, Saint Croix, Venezuela, Cuba and Belize (Márquez et al. 2006, Molinari et al. 2023, Morejón-Arrojo and Rodríguez-Viera 2025). *Nausithoe punctata* can be distinguished from the closely-related *N. maculata* by the absence of pigmentation in the lappets and its larger body size (Molinari et al. 2023).

#### Nausithoe rubra

Vanhöffen, 1902

A8F7CA4A-6164-50FB-8ACD-49FFB92E0A9F

https://www.marinespecies.org/aphia.php?p=taxdetails&id=135291

##### Distribution

See Fig. 1, Mendoza-Becerril et al. (2025).

##### Notes

*Nausithoe rubra* was recorded twice in the Mexican Caribbean; one record came from plankton net samples collected during an oceanographic cruise at depths of 0–200 m (Segura-Puertas and Ordóñez-López 1994) and the other from shallow coastal waters (< 10 m) (Tovar-Juárez et al. 2025). Outside the Mexican Caribbean, *N. rubra* has been reported from Peru — depth not reported — California and the Galápagos Islands, with the latter two collected from depths greater than 300 m, suggesting a broad deep-sea distribution (Molinari et al. (2023). The shallow-water record, therefore, represents an extreme departure from the known depth range of *N. rubra*. This discrepancy suggests: (1) misidentification of the shallow specimen, (2) vertical migration or exceptional upwelling or (3) cryptic species requiring molecular separation. As confirmed records of *N. rubra* are predominantly from the Pacific Ocean, the Caribbean record, as well as records from adjacent regions, such as the Gulf of Mexico, should be interpreted with caution. In the absence of voucher material or molecular data from the Mexican Caribbean, the shallow-water record is considered highly questionable and should be confirmed through future collections.

#### Nausithoe sp.

CE9AE9F1-8340-53A5-9DF1-3A29018CF1A4

##### Distribution

See Fig. 1, Mendoza-Becerril et al. (2025).

##### Notes

This category includes a single citizen-science observation lacking sufficient photographic detail for species-level identification. The photograph shows characters consistent with *Nausithoe*, but cannot be matched to known regional species. While this could represent an additional species, insufficient image quality is a more conservative explanation. Future observations with clear photos of diagnostic features (lappet morphology, colouration, rhopalial arrangement) are needed.

#### Periphylla periphylla

(Péron & Lesueur, 1810)

F85EB235-03DA-57C6-BEE9-24377DE7515B

https://www.marinespecies.org/aphia.php?p=taxdetails&id=135294

##### Distribution

See Fig. 1, Mendoza-Becerril et al. (2025).

##### Notes

The specimen examined shows the diagnostic features of the genus *Periphylla*: 12 tentacles, 16 marginal lappets, a dome-shaped exumbrella and a deep annular furrow (Mayer 1910). This species is the only valid member of the genus following a complex taxonomic history in which *P. hyacinthina* Haeckel, 1880, *P. humilis* Fewkes, 1886 and *P. regina* Haeckel, 1880 were synonymised (Collins et al. 2025). *Periphylla periphylla* has been recorded in several deep sea areas, such as the Gulf of St. Lawrence, the Strait of Belle Isle and southwards to the coast of Virginia (Collins et al. 2025). Additional records document its presence in the Gulf of Mexico at depths exceeding 600 m (Lucas 2008, Lucas and Reed 2009). Other reports show its presence in northern and southern polar waters, the Pacific, Indian and Mediterranean seas (see Båmstedt 2023). This global distribution supports the identification of our specimen from the Caribbean Sea as *P. periphylla* (Fig. 2c).

#### Discomedusae

Haeckel, 1880

79A6CF8D-2F58-5F32-BFCA-38A51337839C

#### Rhizostomeae

Cuvier, 1800

27B8E4EB-1357-5021-9300-042B031E8AAB

#### Cassiopeidae

Tilesius, 1831

398EA460-F0E7-5D28-A244-D70B50B54468

#### Cassiopea andromeda

(Forskål, 1775)

E71C7A33-5439-5339-A65F-718F93A33B36

https://www.marinespecies.org/aphia.php?p=taxdetails&id=135295

##### Distribution

**Figure 3.**
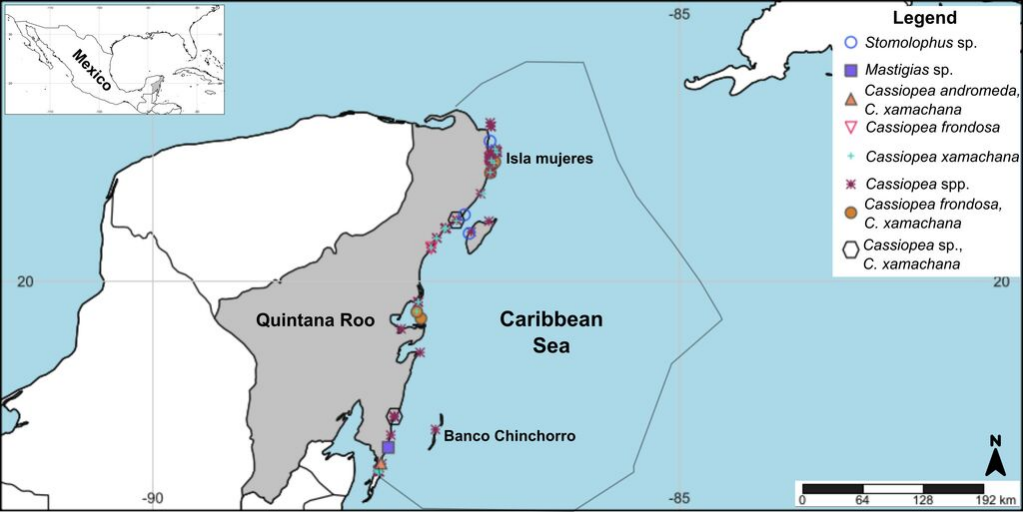
Literature, citizen science and samples collected records of Scyphozoa, Rhizostomeae in the Mexican Caribbean from 1985 to 2024. *Cassiopea* spp. indicates records identified only to genus level. Each symbol represents a single observation at a given locality; the use of “spp.” reflects uncertainty regarding species identity across the dataset rather than the presence of multiple species at individual sites.

See Fig. 3, Mendoza-Becerril et al. (2025).

##### Notes

*Cassiopea andromeda* was originally described from the Suez Canal, Red Sea (Mayer 1910); therefore, this lineage is not naturally distributed in the Caribbean Sea as *C. xamachana* and *C. frondosa* are. In the Mexican Caribbean, *C. andromeda* was first reported by Gamero-Mora (2014), based on four specimens collected at a single locality in Xcalak, Quintana Roo. Even though these individuals were initially misidentified as *C. frondosa* (Pallas, 1774) (likely due to similarities in the morphology of the oral appendages), it is possible to confirm, based on the phylogenetic hypothesis provided by the author, that they belong to *C. andromeda*. Since that report, no additional confirmed records of *C. andromeda* have been documented from the region.

A recent molecular study shows that *C. andromeda* co-occurs with *C. xamachana* at three sites in the Florida Keys, USA, with phenotypical overlap between both species (Muffett and Miglietta 2023), suggesting that its presence in Greater Caribbean may be underestimated. At present, there is no evidence indicating whether *C. andromeda* is established in the Mexican Caribbean or represents a rare occurrence. Given the limited available data and the taxonomic complexity of the genus, continued monitoring and molecular surveys are recommended. For complementary information of morphological similarity and identification challenges, see taxonomic remarks under *C. xamachana*.

#### Cassiopea frondosa

(Pallas, 1774)

C40527B8-FBC8-55F1-9F1C-F2FC02D6CCB4

https://www.marinespecies.org/aphia.php?p=taxdetails&id=287166

##### Distribution

See Fig. 3, Mendoza-Becerril et al. (2025).

##### Notes

This species is the most morphologically distinctive member of the genus, readily identified by the presence of 12 rhopalia and by its oral appendages, which are flattened and leaf-shaped (Mayer 1910); this diagnostic character is visible in the citizen-science record shown in Fig. 2d. These characters distinguish *C. frondosa* from other *Cassiopea* species reported from the Mexican Caribbean, which lack flattened, leaf-shaped appendages, show greater variation in appendage colour and shape and typically possess around 16 rhopalia. Previous records by Collado et al. (1988) and subsequent studies reporting this species in the Mexican Caribbean were likely correct, based on these same traits. The presence of *C. frondosa* in the Mexican Caribbean is, therefore, accepted as valid and the name is retained in this checklist (Fig. 2d).

#### Cassiopea xamachana

Bigelow, 1892

90FD423E-CDC1-5119-9D29-B91C135C20C7

https://www.marinespecies.org/aphia.php?p=taxdetails&id=287172

##### Distribution

See Fig. 3, Mendoza-Becerril et al. (2025).

##### Notes

*Cassiopea xamachana* and *C. andromeda* have been described as morphologically similar and even indistinguishable species (Hummelinck 1968). Further evidence of sympatry comes from Florida, where *C. xamachana* and *C. andromeda* have been recorded co-occurring at the same localities across multiple sites (Muffett and Miglietta 2023), although *C. andromeda* was first described from the Red Sea. At those sites in Florida, individuals of both species show overlapping morphology and colouration, with no consistent external traits to distinguish them reliably (Fig. 2e).

Five specimens from the Mexican Caribbean were sequenced in this study for the 16S marker and showed 99.63–100% similarity with sequences of *C. xamachana* from Florida and Panama and 96.88% similarity with *C. andromeda* from Florida (Suppl. material 1). These values fall within the interspecific divergence ranges reported for *Cassiopea* using 16S (Gamero-Mora et al. 2022). Although based on a limited number of specimens and a single mitochondrial marker, these results confirm the presence and predominance of *C. xamachana* in the region and are consistent with previous morphological records indicating the species’ presence in the Mexican Caribbean.

In citizen-science records from the Mexican Caribbean included in this checklist, individuals were originally reported as *C. xamachana* by contributors and community review and consistently show brightly-coloured oral appendages, a trait commonly associated with the species in the region. Unlike *C. xamachana*, for which colour variation has been documented at a single locality and whose consistency across populations remains unknown, *C. andromeda* usually lacks this pigmentation in the Mexican Caribbean (Gamero-Mora 2014). However, Muffett and Miglietta (2023) later showed that colouration in *Cassiopea* is highly variable and not species-specific. For this reason, colouration patterns should be interpreted with caution and should never be used alone for species identification without genetic or robust morphological verification. Accordingly, the presence of *C. andromeda* in the Mexican Caribbean may be underestimated in citizen-science records.

#### Cassiopea spp.

AAB9C26A-9138-54FC-B92F-1D857123A750

##### Distribution

See Fig. 3, Mendoza-Becerril et al. (2025).

##### Notes

This category includes records identified as *Cassiopea* sp., most of them being based on citizen-science photographs. Many of these image records only show the exumbrellar view or a limited oral perspective which makes it difficult to see key diagnostic features, such as the number of rhopalia or the shape and colour of the oral appendages. As *Cassiopea* species are mainly distinguished by oral structures (Gamero-Mora et al. 2022), it was not possible to assign them to a particular species. They may correspond to *C. xamachana*, *C. andromeda* or even *C. frondosa*, yet, in the absence of clearer photos or physical material, they are conservatively listed as *Cassiopea* spp. As these records likely represent observations of species already documented for the region, they were not included in species richness counts.

#### Stomolophidae

Haeckel, 1880

9DC01DB1-0611-5D9F-8047-A233FF4DAEF0

#### Stomolophus ssp.

7E61A3B4-35E8-51DA-9DF9-0F0A86365145

##### Distribution

See Fig. 3, Mendoza-Becerril et al. (2025).

##### Notes

On the Atlantic coast of Mexico, several records have referred to *S. meleagris* Agassiz, 1860, but in this study, all occurrences from the Mexican Caribbean are reported simply as *Stomolophus* sp. This decision follows the lack of voucher specimens. Molecular data confirm *S. meleagris* in the Gulf of Mexico on both the Mexican side (Tabasco) and the U.S. coast, including Florida and Dauphin Island (Bayha and Dawson 2010, Gómez Daglio and Dawson 2017). Other genetic lineages have been reported in nearby areas. One example is *Stomolophus* sp. 5, recorded from the Caribbean coast of Nicaragua by Gómez Daglio and Dawson (2017), suggesting that more than one species of the genus may occur in the wider region. Furthermore, specimens from northern South America have been identified as *S. fritillaria* Haeckel, 1880 (Kramp 1955); taking this into account and due to the lack of molecular data for the Mexican Caribbean, all records are conservatively listed as *Stomolophus* sp. until further studies clarify species boundaries (Fig. 2f).

#### Mastigiidae

Stiasny, 1920

038E4468-A163-5778-A482-D9DE2B6A8E47

#### Mastigias sp.

2FB991BC-505B-5D01-BA04-226B079EEFBB

##### Distribution

**Figure 4.**
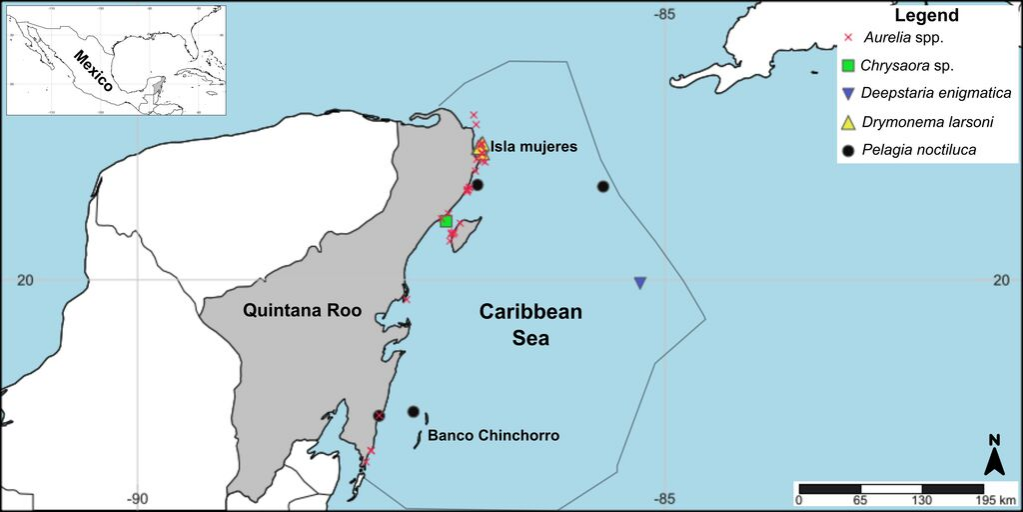
Literature, citizen science and samples collected records of Scyphozoa, Semaeostomeae in the Mexican Caribbean from 1972 to 2024. *Aurelia* spp. indicates records identified only to genus level. Each symbol represents a single observation at a given locality; the use of “spp.” reflects uncertainty regarding species identity across the dataset rather than the presence of multiple species at individual sites.

See Fig. 3Fig. 4, Mendoza-Becerril et al. (2025).

##### Notes

The specimen examined shows the typical morphological characteristics of the genus *Mastigias* (Mayer 1910), including a hemispherical bell, absence of marginal tentacles and eight oral arms bearing three wing-like extensions along their length that terminate in club-shaped projections. In life, the medusa exhibited a light brown bell with conspicuous circular white spots. The specimen had a bell diameter of 10.5 cm.

The individual was collected in Río Huache, Quintana Roo, Mexican Caribbean and is housed in ECOSUR, ZL (Table 1) . Although species-level identification in this genus is difficult due to variation in colouration, exumbrellar patterning and body proportions (de Souza and Dawson (2018), our specimen was identified as *Mastigias* sp. 1. According to de Souza and Dawson (2018), jellyfish of this genus are naturally distributed across the tropical and subtropical Indo-Pacific, from the Fijian Islands to the western Indian Ocean and from Japan to Australia. However, non-native records have been reported in Hawaii (Eldredge and Smith 2001) and in the Caribbean and Gulf of Mexico (Bayha and Graham 2011, Bayha and Graham 2014). Molecular analyses using mitochondrial markers of specimens from Puerto Rico and Florida showed they are genetically closest to *Mastigias* sp. 1 from Indonesia (see Bayha and Graham (2011)). To date, *Mastigias* sp. 1 is the only lineage reported from the Caribbean Region and the record presented here represents the first and only confirmed occurrence of the genus in the Mexican Caribbean (Fig. 5a).

#### Semaeostomeae

Agassiz, 1862

E09644A4-737E-5647-9201-8E3D4316100B

#### Drymonematidae

Haeckel, 1880

032D769B-0F73-5C04-9AB8-24149BF8105D

#### Drymonema larsoni

Bayha & Dawson, 2010

C6A91FA3-8AE5-5C95-A05F-D364552A12B7

https://www.marinespecies.org/aphia.php?p=taxdetails&id=867502

##### Distribution

See Fig. 4, Mendoza-Becerril et al. (2025)

##### Notes

The records included here are based on citizen-science observations. Two of the three valid species of *Drymonema* have been reported from the western Atlantic: *D. gorgo* Müller 1883 and *D. larsoni* (Collins et al. (2025). *Drymonema gorgo* was described from Brazil and has also been recorded in the Caribbean and the south-western Atlantic, including Bermuda and Argentina (Bayha and Dawson 2010, Gómez Daglio and Dawson 2017). *Drymonema larsoni*, on the other hand, was described from Dauphin Island, Alabama, with molecular confirmation from the type locality and from Florida (Bayha and Dawson 2010, Gómez Daglio and Dawson 2017). Paratypes from the British Virgin Islands and Puerto Rico support its presence in the Caribbean and additional records from Bermuda and South Carolina indicate a broad western Atlantic distribution (Bayha and Dawson 2010). As the records analysed here come from the Mexican Caribbean, where *D. larsoni* is the only species confirmed by morphological and molecular data, all observations are retained under that name in this checklist.

#### Pelagiidae

Gegenbaur, 1856

3A4D57E4-980D-51E6-9904-04DEE78F8387

#### Chrysaora sp.

478E8E5A-345E-50B8-8ABF-3EAF2D5F7281

##### Distribution

See Fig. 4, Mendoza-Becerril et al. (2025).

##### Notes

Specimens were assigned to the genus *Chrysaora*, based on diagnostic characters of pelagiid scyphomedusae (Morandini and Marques 2010), including a hemispherical bell, three marginal tentacles per octant (24 in total) between successive pairs of marginal sense organs and eight elongate, frilled oral arms.

On the Atlantic coast of Mexico, *Chrysaora quinquecirrha* (Desor, 1848) has been reported in coastal waters of Tabasco (Gómez Daglio and Dawson 2017). Both *C. quinquecirrha* and *C. chesapeakei* (Papenfuss, 1936) have also been recorded in the northern Gulf of Mexico along the U.S. coast (Bayha et al. 2017). In the Gulf of Mexico and the Caribbean, *C. lactea* Eschscholtz, 1829 has been mentioned, based on morphological features, but its taxonomic status remains uncertain (Morandini and Marques 2010). Another lineage, *Chrysaora* sp. 6, sensu Gómez Daglio and Dawson (2017), was discovered along the Caribbean coasts of Panama and Costa Rica suggesting the presence of undescribed diversity in nearby regions. Regional currents promote connections amongst the Gulf of Mexico, Central America and the Mexican Caribbean (Murphy et al. 1999) and this oceanographic continuity could explain the occurrence of multiple *Chrysaora* species in the region. Considering these uncertainties, we report all records as *Chrysaora* sp. until future integrative studies clarify their identities (Fig. 5b, c).

#### Pelagia noctiluca

(Forsskål, 1775)

28957F52-AC2F-5638-BEDD-B888EB2E9A66

https://www.marinespecies.org/aphia.php?p=taxdetails&id=135305

##### Distribution

See Fig. 4, Mendoza-Becerril et al. (2025).

##### Notes

Five valid species are currently recognised within the genus *Pelagia*: *P. noctiluca*, *P. cyanella* Péron & Lesueur, 1810, *P. discoidea* Eschscholtz, 1829, *P. flaveola* Eschscholtz, 1829 and *P. panopyra* Péron & Lesueur, 1810 (Collins et al. 2025). However, all species, except *P. noctiluca*, are considered “species requiring further studies, doubtful” which highlights the uncertain taxonomy of the group. *Pelagia noctiluca* is regarded as a species found across many temperate and tropical regions making its presence in the Caribbean plausible. Gómez Daglio and Dawson (2017) discovered a distinct Caribbean lineage, *Pelagia* sp. 1, from Isla Margarita, Venezuela. Although Gómez-Daglio and Dawson (2017) included *Pelagia* sp. 1 in their morphological dataset, the PCA results were explicitly reported only for the comparison between *P. noctiluca* and *P. panopyra* and no morphological distinctions between *Pelagia* sp. 1 and *P. noctiluca* were reported. Considering the oceanographic connectivity in the region, it is possible that this lineage extends into the Mexican Caribbean. Accordingly, although *P. noctiluca* has been previously confirmed in the Caribbean, the records reported here are treated as tentative assignments and could alternatively correspond to *Pelagia* sp. 1 or another unresolved regional lineage, pending molecular confirmation (Fig. 5d).

#### Ulmaridae

Haeckel, 1880

417E1BB9-FEA8-5C49-960F-413CD783C233

#### Aurelia spp.

FADA8A43-2F42-5F3A-80B0-CA9A2269A287

##### Distribution

See Fig. 4, Mendoza-Becerril et al. (2025).

##### Notes

Although some records from literature, the citizen-science platform iNaturalist and samples collected have referred to *Aurelia* species, using names such as *A. aurita* (Linnaeus, 1758), *A. marginalis* Agassiz, 1862 or *A. coerulea* von Lendenfeld, 1884, all occurrences from the Mexican Caribbean are here reported simply as *Aurelia* sp. However, the use of Aurelia spp. in this section reflects the likelihood that multiple species of *Aurelia* are present in the region. This decision was made because of the absence of voucher specimens and on the well-known difficulty of distinguishing species within this genus, using only morphological data. As noted by Lawley et al. (2021), morphological variability in *Aurelia* often obscures diagnostic boundaries, making reliable identification particularly challenging without molecular data. Accordingly, these records are counted as a single species in the checklist, although they may represent more than one species.

*Aurelia aurita* and *A. coerulea* have been confirmed genetically in north-eastern North America and along the Atlantic coast of Europe, but molecular data for these species are still lacking in the Mexican Caribbean. In nearby regions, at least six other *Aurelia* species have been identified: *A. insularia* Lawley et al., 2021 (Key Largo, Florida), *A. marginalis* (Tabasco, Mexico; Alabama and Florida, USA), *A. montyi* Lawley et al., 2021 and *A. rara* Lawley et al., 2021 (Alabama and Florida, USA), *A. smithsoniana* Lawley et al., 2021 (Bocas del Toro, Panama) and *Aurelia* sp. 18 (Louisiana, USA) (Lawley et al. 2021). Some of these species even occur together in the same areas, possibly due to ocean currents that connect Central America, the Gulf of Mexico and the Mexican Caribbean (Murphy et al. 1999) and may facilitate the transport of medusae or planulae across regions. Given these regional connections and the unresolved taxonomy of the genus, the records are reported here as *Aurelia* sp. (Fig. 5e, f).

#### Deepstaria enigmatica

Russell, 1967

CFC7CE40-C2FA-503C-B80E-5B4B31A8CDE4

https://www.marinespecies.org/aphia.php?p=taxdetails&id=135307

##### Distribution

See Fig. 4, Mendoza-Becerril et al. (2025).

##### Notes

This species was reported from the Mexican Caribbean by Phillips (1972), based on a single damaged female specimen with a bell diameter of more than 70 cm. The depth at which the specimen was collected could not be determined with certainty, as the sampling device fished from the surface to approximately 2400 m without the use of a closing mechanism (Phillips 1972). At present, two valid species are recognised within the genus: *Deepstaria enigmatica*, originally described from San Diego in the Pacific Ocean and *D. reticulum* Larson, Madin & Harbison, 1988 from Bermuda in the Atlantic Ocean. At the time of Phillips’s report, *D. reticulum* had not yet been described. *Deepstaria enigmatica* has been recorded far from its type locality, including deep-sea observations from the mid-North Atlantic obtained using ROVs (Gruber et al. 2018), but the Mexican Caribbean record lies closer to the type locality of *D. reticulum*, suggesting that the specimen observed by Phillips may actually belong to that species. However, geographic proximity alone is insufficient for re-assignment in the absence of diagnostic morphological evidence. Given the absence of voucher material, the uncertainty regarding collection depth and the lack of diagnostic morphological information available for re-examination, the identification cannot be re-assessed with confidence. We, therefore, retain the original identification as *D. enigmatica*, while explicitly acknowledging that this record should be regarded as provisional.

## Analysis

A total of 128 records were compiled from literature sources, corresponding to 33 references, of which 30 are scientific articles or book chapters and three are theses of different academic levels. Sixty-four percent of these publications were published between 1990 and 2010. Of the total records, six were not considered for analysis of distribution and taxa recorded because four corresponded to compilations and two to aquarium studies. Therefore, 122 records were considered, of which 118 corresponded to species level, three to genus level and one to class level; the records total 10 species (Table 2).

A total of 221 records were collected from citizen-science between 2003 and 2025. Of these, 84% were observations made by iNaturalist users. Of the total records, 65 were determined at the species level, 152 at the genus level and four at higher categories (Mendoza-Becerril et al. 2025). Using this source of information, we identified five species (Table 2).

From the samples collected between 1987 and 2024, a total of 35 records were obtained, of which 32 were identified to species level and three to genus level. Recording six species identified to species level with two additional records identified only to genus level (Table 2).

Of the taxa identified to genus and species level, five were only recorded in literature, four were recorded only through citizen-science and two came exclusively from collected samples. In contrast, only four taxa were present in all three sources of information analysed (Table 2).

## Discussion

Mexico hosts 35 documented species of Scyphozoa belonging to 16 families, but only eight species were previously recorded from the Mexican Caribbean (Gasca and Loman-Ramos 2014, Estrada-González et al. 2023). In the present study, the number of species observed in this region increased to 17, including nine new records. Amongst them are the non-native species *Cassiopea andromeda* and *Mastigias* sp. Their detection underscores the value of integrative inventories for recognising introduced taxa, which may otherwise remain overlooked in regionally focused or single-source assessments, despite their potential to affect native communities once established. This increase reflects the advantage of combining multiple sources of information (literature, citizen science and collected specimens), which together provide a more comprehensive view of regional diversity than any single approach alone (see also Morejón-Arrojo and Rodríguez-Viera (2025)). The integration of these complementary datasets reveals that the richness of Scyphozoa in the Mexican Caribbean has been underestimated, highlighting the importance of multi-source inventories to refine biodiversity assessments and detect both native and introduced species.

*Cassiopea xamachana* and *Aurelia* spp. (134 records) represented most of the records in this study, which reflects their widespread distribution in the region. In this regard, both species were commonly found in shallow coastal environments, incuding lagoons, which is consistent with the fact that both taxa are known for their wide distribution across temperate and tropical waters worldwide (Gómez-Aguirre et al. 2018, Ohdera et al. 2018). The high frequency of *C. xamachana* records may be explained by its semi-benthic habit, which keeps the medusae close to the substrate and makes them easier to observe in calm, shallow waters. In addition, as members of the genus harbour photosynthetic symbiotic algae, they are typically restricted to shallow, clear waters where light penetration is sufficient. This lifestyle, combined with its tolerance to variations in temperature and salinity, allows the species to thrive in coastal lagoons and disturbed habitats (Ohdera et al. 2018). These traits, together with its association with photosynthetic symbiotic algae, also position *C. xamachana* as a potential bioindicator species for degraded or anthropogenically influenced environments, particularly those affected by urban and tourist activities (Stoner et al. 2011, Stoner 2014). This ecological versatility, combined with the species’ conspicuous behaviour and habitat preferences, could explain its prevalence in both field surveys and citizen-science records, which highlights its importance in monitoring environmental changes in the coastal ecosystems of the Mexican Caribbean.

Citizen science proved to be a valuable approach for documenting Scyphozoa species richness in the study area, consistent with approaches previously applied to cubozoans (see Villalobos-León and Mendoza-Becerril (2025) and in this study yielded more than two hundred observations compiled through the iNaturalist platform, thereby expanding the known distribution of several species. Notably, large medusae, such as *D. larsoni*, were recorded only through this method, probably because their size and fragility make them difficult to collect, preserve and study through laboratory or field-based research. Similar initiatives have shown the importance of citizen participation in marine biodiversity studies. In this regard, Puente-Tapia et al. (2021) used the iNaturalist network to update the list of ctenophores in Mexican waters and Rocha et al. (2024) demostrated that iNaturalist provides reliable data for many marine taxa when validated by experts. Nevertheless, citizen-science data are subject to observational and taxonomic biases, particularly favouring large, conspicuous species and often limiting identification to genus level. Together, our findings and previous evidence confirm that citizen science can reveal taxa overlooked by conventional sampling and emphasise the importance of expert review, as implemented here through systematic evaluation of all records by the authors (see Material and methods section), to ensure accurate species identification; thus, including citizen-science observations in this checklist was essential for obtaining a more complete view of jellyfish species richness in the study region.

Our dataset included a few records of the *Nausithoe* species from citizen-science data; however, these observations differ from typical public contributions. A specialised diving company (Blackwater Cozumel Scuba Diving Centre) generated most of these records; thus, although these records are interesting, they do not represent conventional citizen-science observations in the sense of public participation. In line with this, Morejón-Arrojo and Rodríguez-Viera (2025) reported occurrences of *Nausithoe*, *Atolla* and *Linuche* obtained from OBIS and/or scientific literature, both of which compile data from oceanographic expeditions and institutional research rather than from public participation. Therefore, the majority of *Nausithoe* records in the Mexican Caribbean derive from professional studies and plankton surveys conducted by universities or research programmes (Phillips 1972, Segura-Puertas and Ordóñez-López 1994, Suarez-Morales et al. 1999), highlighting that these deep-water medusae are documented primarily through formal research efforts rather than citizen-science initiatives.

All records analysed in this study correspond to the medusa stage, with no evidence of other life cycle phases, such as polyps or ephyrae. As shown in Mendoza-Becerril et al. (2025), most records were concentrated in specific months of the year, coinciding with periods of greater sampling activity and jellyfish abundance. This pattern indicates both temporal and methodological biases. The predominance of medusa records likely reflects the higher visibility and easier recognition of this stage (Lawley et al. 2021), particularly in citizen-science photographs and plankton surveys, whereas benthic stages are small, cryptic and rarely targeted in fieldwork. In the case of citizen-science data, records may reflect vacation or tourism periods, during which observers spend more time in coastal areas and are more likely to document visible organisms, such as jellyfish. Similar temporal biases have already been demonstrated for citizen-science data, where recording activity increases during mild weather, during spring and on weekends (Rosário et al. 2025). Additionally, records from aquarium observations (41 records) were excluded from our analyses because their origin could not be independently verified. Consequently, the dataset presented here probably captures only part of the phenological and ecological variability of Scyphozoa in the Mexican Caribbean, highlighting the need for complementary studies, focused on benthic and early developmental stages to achieve a more complete understanding of their life cycles and ecological dynamics (Lucas 2001).

The strong bias toward medusa-stage records in this study has important implications for our understanding of true jellyfish biodiversity and ecology in the Mexican Caribbean. Benthic stages of scyphozoans, such as polyps, represent the long-term resident phase of the life cycle and play a central role in jellyfish population dynamics (Lucas 2001). The absence of information on polyps limits the interpretation of species persistence and spatial structure in the Mexican Caribbean. Consequently, species checklists that rely on medusa records provide an incomplete view of Scyphozoa species richness and make evident the need for integrative studies that explicitly include benthic and early life stages.

Several taxa in this checklist were identified only to the genus level, such as *Chrysaora* sp. and *Stomolophus* sp., due to the lack of voucher specimens or the high morphological similarity amongst regional lineages. Additionally, molecular studies in nearby regions have revealed the existence of distinct, but undescribed lineages within both genera; for example, along the Caribbean coast, *Stomolophus* sp. 5 was reported in Nicaragua and *Chrysaora* sp. 6 in Panama and Costa Rica (Gómez Daglio and Dawson 2017). These findings suggest that the western Atlantic and Caribbean harbour a complex mosaic of closely-related lineages whose morphological boundaries remain uncertain. Due to the oceanographic connectivity between the Gulf of Mexico and the Mexican and Central American Caribbean (Murphy et al. 1999), it is plausible that some of these lineages extend into the study area. Nonetheless, in the absence of recent morphological revisions for these genera, it is not possible to compare the available material with type material of valid species or against regionally described species in a consistent way; thus; this combination of oceanographic connectivity and unresolved taxonomy highlights the need for integrative approaches that combine morphology and molecular data to clarify species boundaries and assess the true extent of scyphozoan species richness in the Mexican Caribbean.

Studies on the ecology, distribution, abundance and diversity of Scyphozoa in the Mexican Caribbean have focused mainly on shallow environments such as reef lagoons, estuaries and coastal areas (e.g. Segura-Puertas (1992), Segura-Puertas and Ordóñez-López (1994), Segura-Puertas and Damas-Romero (1997), Suarez-Morales et al. (1999), Suárez-Morales et al. (1999), Gasca et al. (2003), Canché-Canché and Castellanos-Osorio (2005)), while studies from oceanic and deep sea zones are scarce; for example, of the data collected in Mendoza-Becerril et al. (2025), 55 records correspond to samples taken at a depth > 20 m, i.e. 17% of the data collected. Thus, this uneven sampling effort, together with the difficulties in identifying certain species, hinders a complete grasp of jellyfish biodiversity in the region. Uncovering a likely hidden diversity requires systematic collections and collection of material suitable for morphological and molecular analyses, which, complemented with citizen science, can help bridge spatial and temporal gaps to achieve a more comprehensive understanding of jellyfish diversity in the Mexican Caribbean.

## Supplementary Material

XML Treatment for Coronamedusae

XML Treatment for Coronatae

XML Treatment for Atollidae

XML Treatment for Atolla vanhoeffeni

XML Treatment for Linuchidae

XML Treatment for Linuche unguiculata

XML Treatment for Nausithoidae

XML Treatment for Nausithoe maculata

XML Treatment for Nausithoe punctata

XML Treatment for Nausithoe rubra

XML Treatment for Nausithoe sp.

XML Treatment for Periphylla periphylla

XML Treatment for Discomedusae

XML Treatment for Rhizostomeae

XML Treatment for Cassiopeidae

XML Treatment for Cassiopea andromeda

XML Treatment for Cassiopea frondosa

XML Treatment for Cassiopea xamachana

XML Treatment for Cassiopea spp.

XML Treatment for Stomolophidae

XML Treatment for Stomolophus ssp.

XML Treatment for Mastigiidae

XML Treatment for Mastigias sp.

XML Treatment for Semaeostomeae

XML Treatment for Drymonematidae

XML Treatment for Drymonema larsoni

XML Treatment for Pelagiidae

XML Treatment for Chrysaora sp.

XML Treatment for Pelagia noctiluca

XML Treatment for Ulmaridae

XML Treatment for Aurelia spp.

XML Treatment for Deepstaria enigmatica

5418466A-9DB9-5FA7-8D9A-686ED7711297 10.3897/BDJ.14.e182574.suppl1 Supplementary material 1 Supplementary file Data type Percentage identity matrix Brief description Percentage identity matrix (16S rRNA) showing pairwise sequence similarity between *Cassiopea xamachana* specimens from Quintana Roo, Mexico and reference sequences of *C. xamachana* and *C. andromeda* from Florida and Panama. File: oo_1439832.xlsx https://binary.pensoft.net/file/1439832 Edgar Gamero-Mora, Iván A. Castellanos-Osorio, Laura Celis, María A. Mendoza-Becerril

## Figures and Tables

**Figure 2a.**
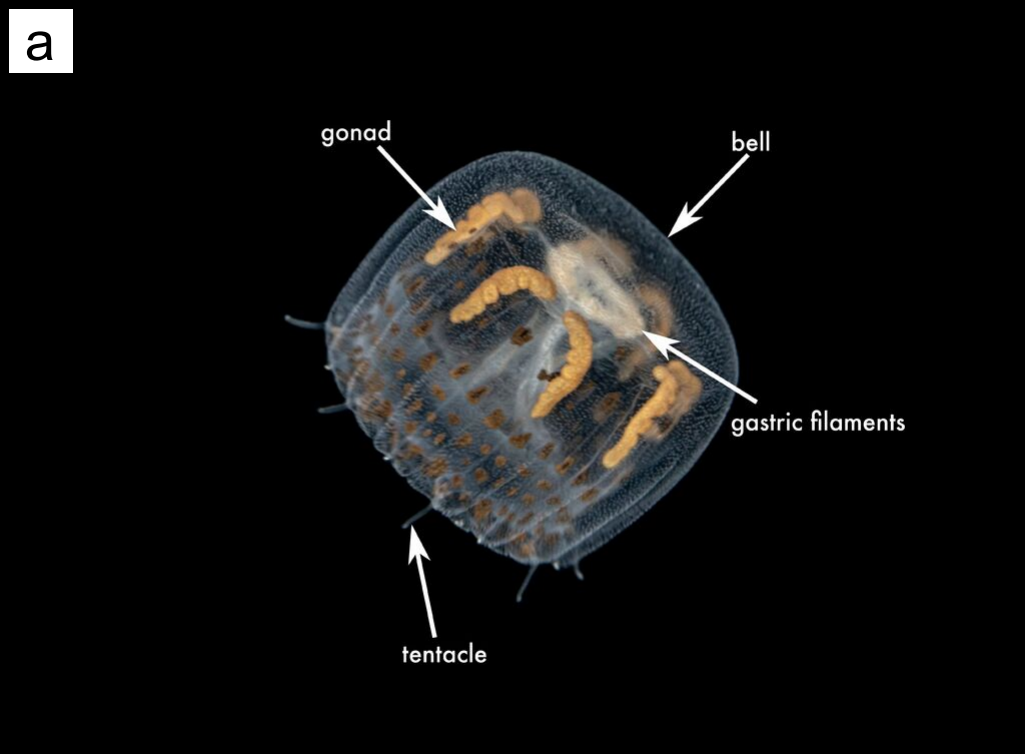
*Linuche unguiculata*;

**Figure 2b.**
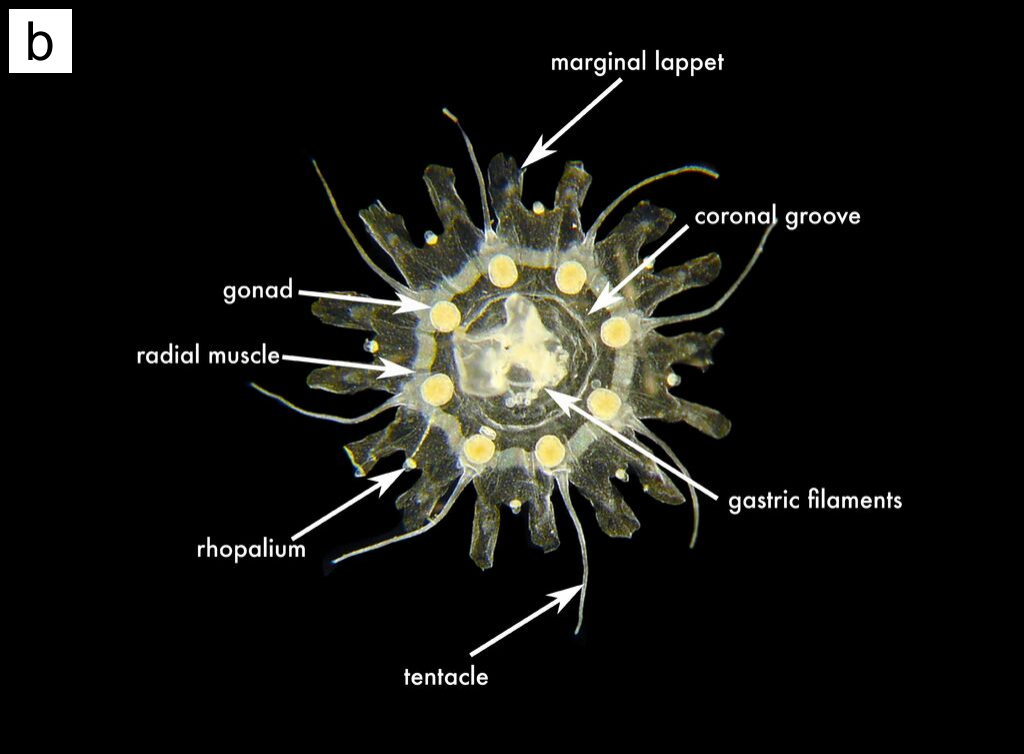
*Nausithoe punctata*;

**Figure 2c.**
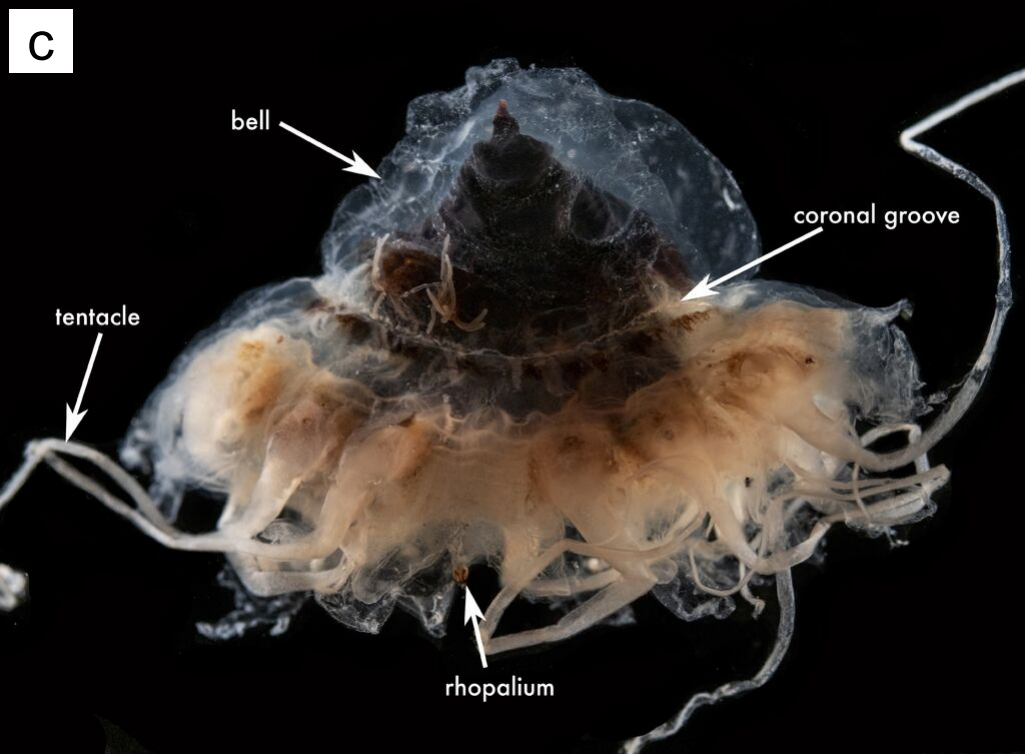
*Periphylla periphylla*;

**Figure 2d.**
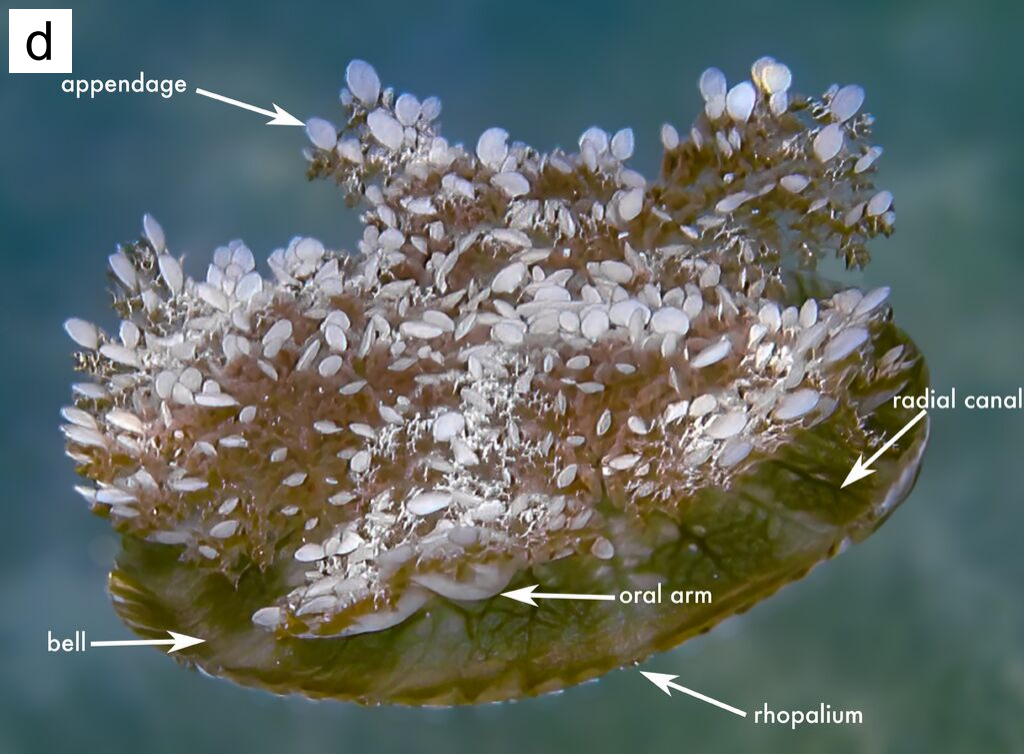
*Cassiopea frondosa*;

**Figure 2e.**
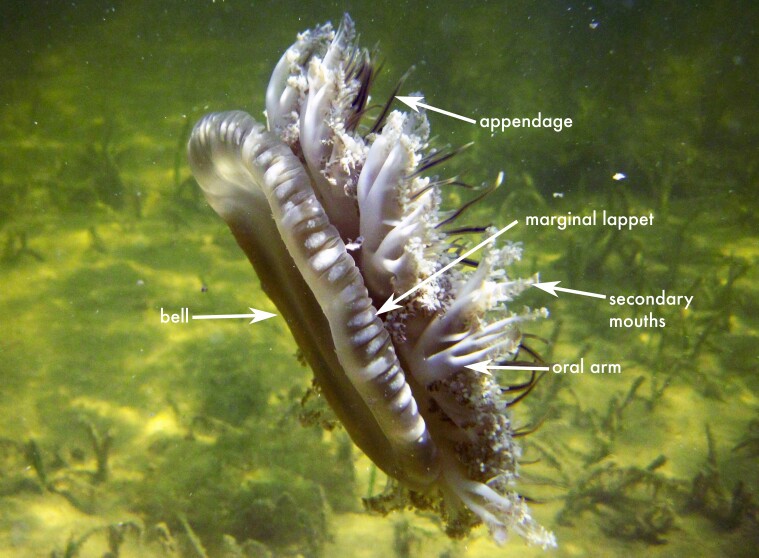
*Cassiopea xamachana*;

**Figure 2f.**
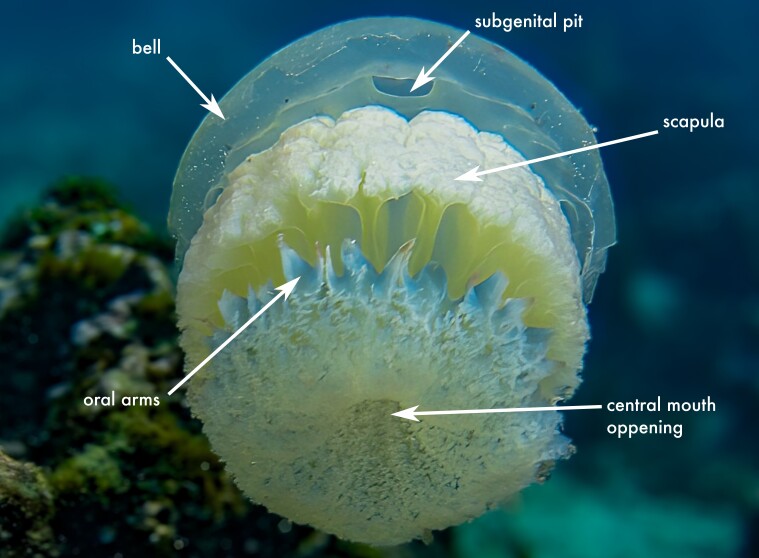
*Stomolophus* sp.

**Figure 5a.**
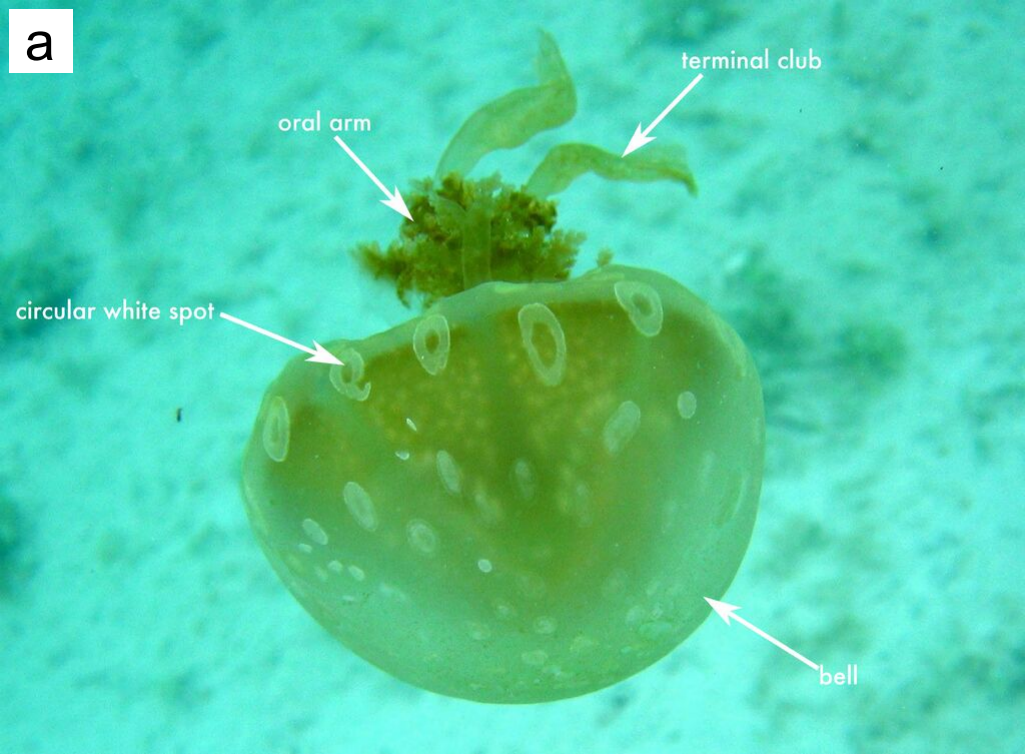
*Mastigias* sp.;

**Figure 5b.**
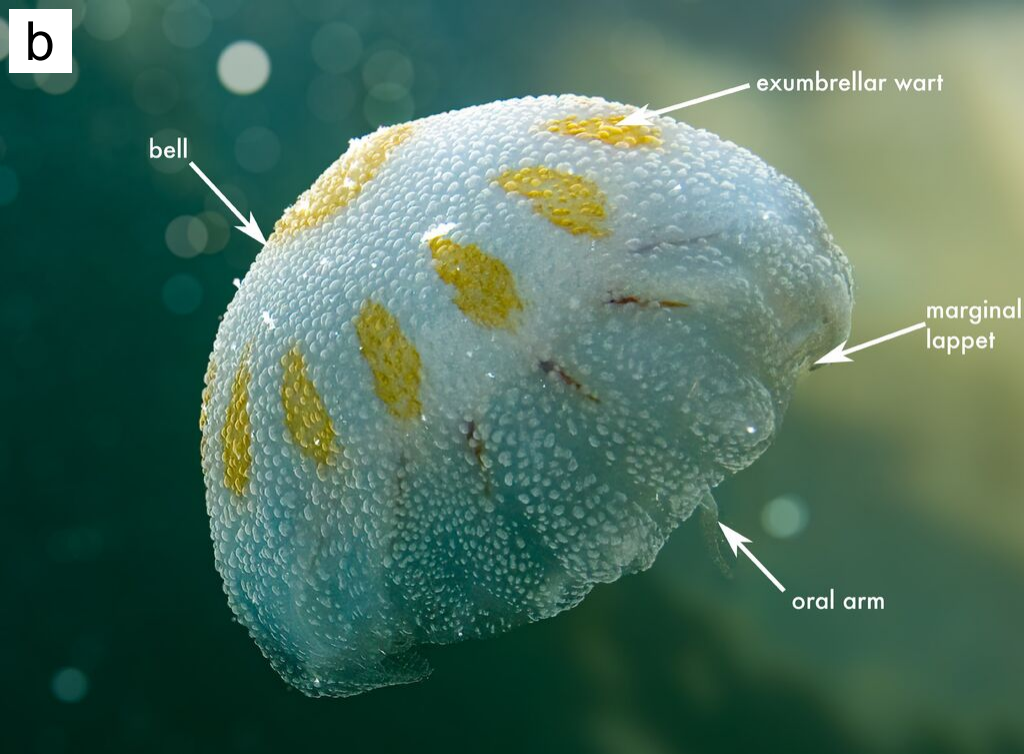
*Chrysaora* sp.;

**Figure 5c.**
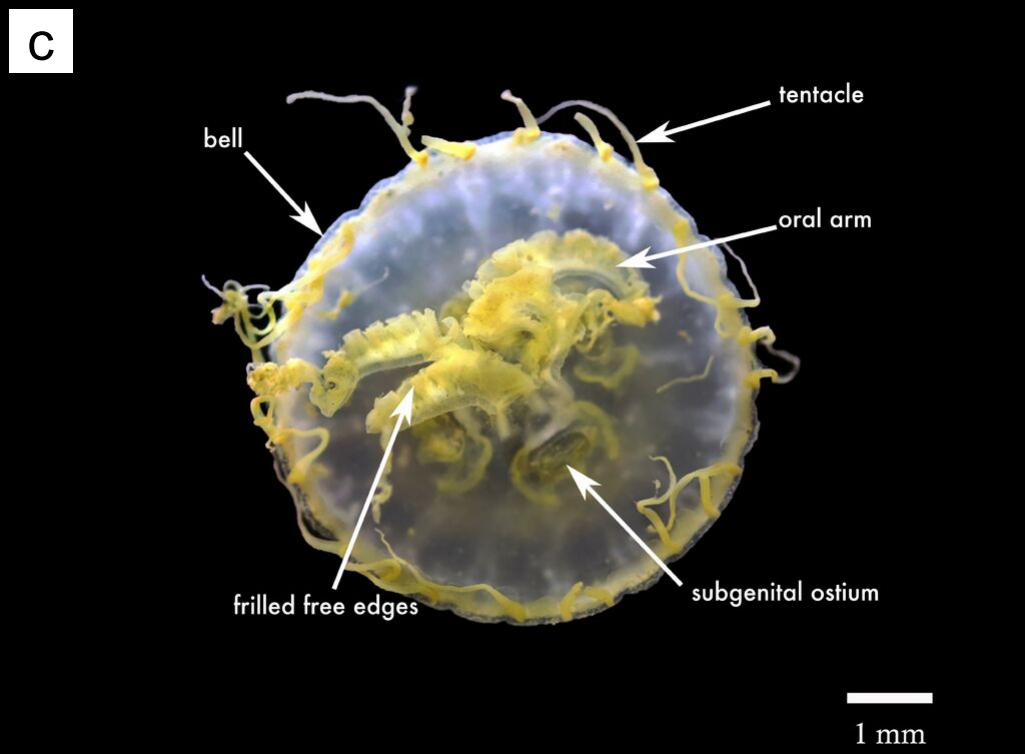
*Chrysaora* sp.;

**Figure 5d.**
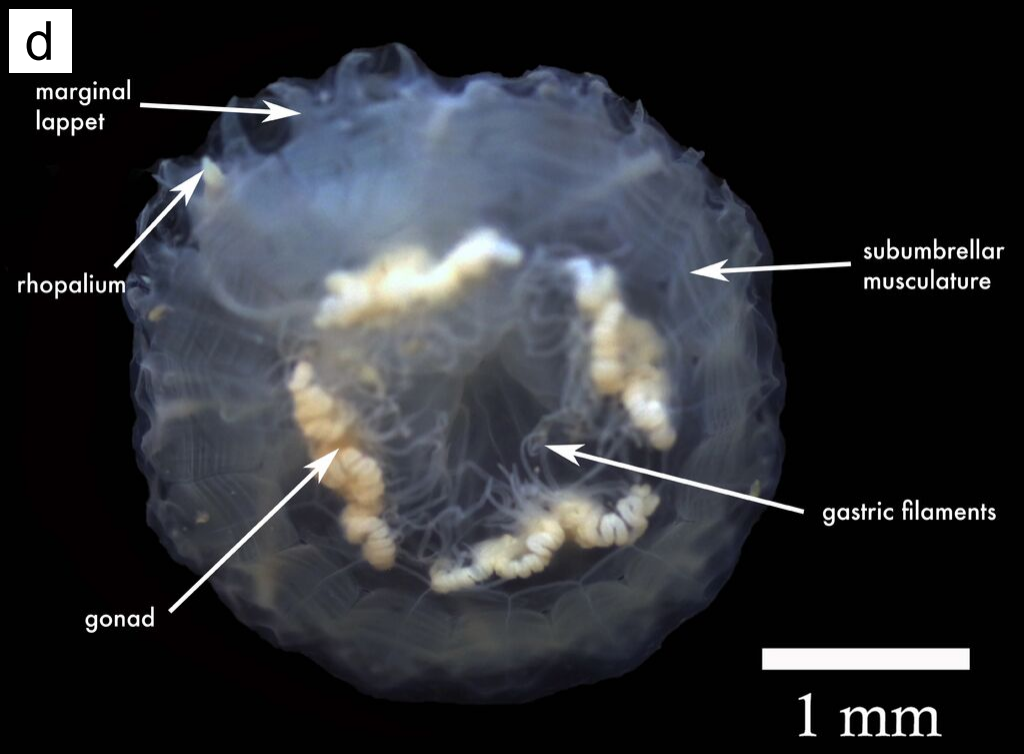
Pelagia noctiluca

**Figure 5e.**
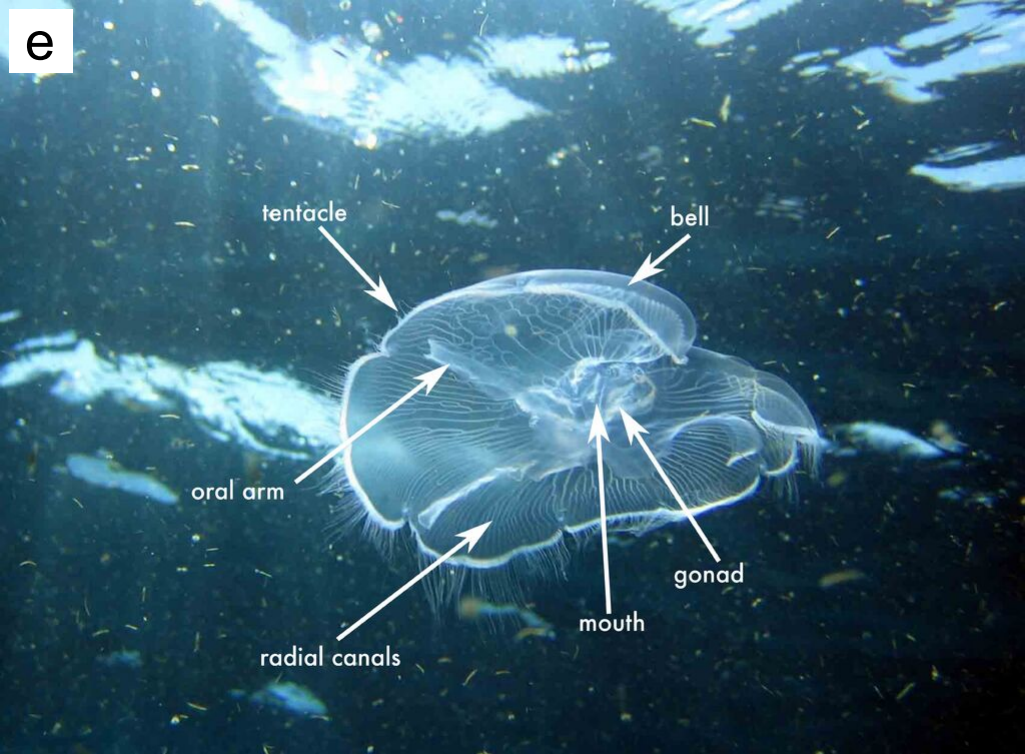
*Aurelia* sp.;

**Figure 5f.**
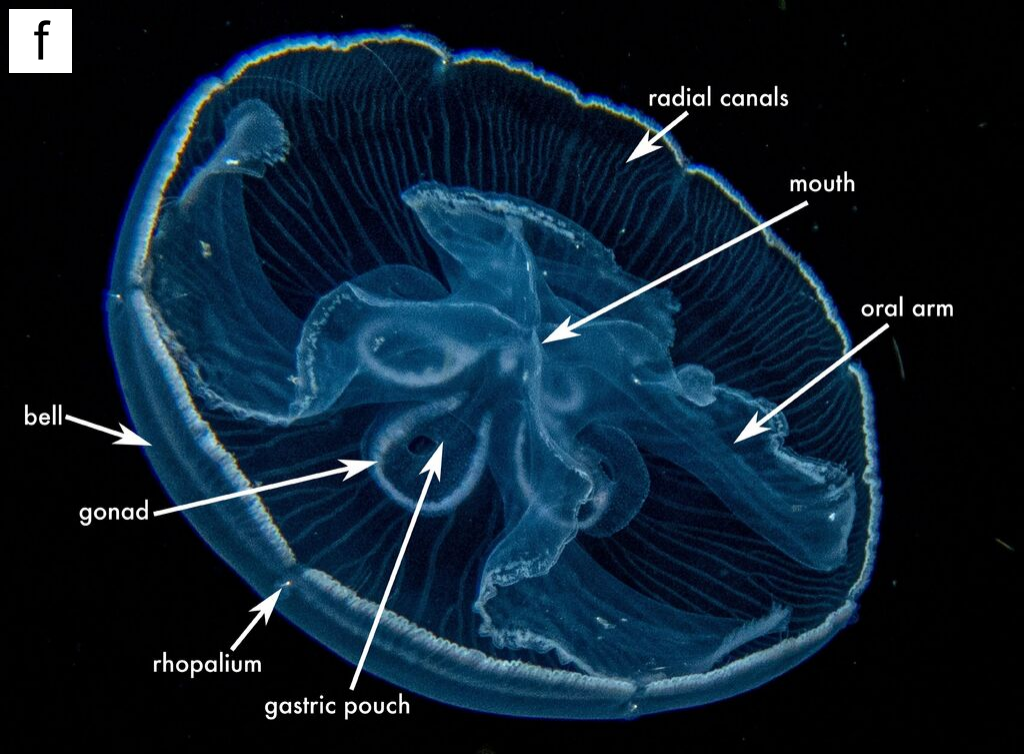
*Aurelia* sp.

**Table 1.** Sampling information for scyphozoan records from the Mexican Caribbean coast. ECOSUR, ZL (El Colegio de la Frontera Sur, Zooplankton laboratory); UNAM, ICML, UASA (Universidad Nacional Autónoma de México, Instituto de Ciencias del Mar y Limnología, Unidad Académica de Sistemas Arrecifales).

**Locality**	**Latitude N**	**Longitude W**	**Sampling method**	**Data (month/year)**	**Depth (m)**	**Fixed sample**	**Institution responsible for holding the samples**
Quintana Roo	No data	No data	Trawl net	01/2007	No data	4% formalin	ECOSUR, ZL
North of Quintana Roo	From 21°06'23" to 20°53'28"	From 86°47'07" to 86°51'13"	Zooplankton net	05/2005	No data	4% formalin	ECOSUR, ZL
Bojórquez lagoon	21°08'50"	86°47'05"	Zooplankton net	05/2005	No data	4% formalin	UNAM, ICMyL, UASA
Nichupté Lagoon	From 21°06'23" to 21°07'55"	From 86°47'07" to 86°46'20"	Zooplankton net	03/2005	No data	4% formalin	UNAM, ICMyL, UASA
	21°01'48"	86°47'41"	Zooplankton net	03/2005	No data	4% formalin	UNAM, ICMyL, UASA
Cayo Culebra	19°42'51.5"	87°29'42.3"	Net with a telescopic handle	07/1987	0-3	4% formalin	ECOSUR, ZL
Chinchorro bank	18°45'00.0"	87°23'00.0"	Net with a telescopic handle	06/2001	No data	4% formalin	ECOSUR, ZL
Río Huache	18°25'17.4"	87°45'54.0"	Net with a telescopic handle	04/2006	No data	4% formalin	ECOSUR, ZL
Xcalak	18°16'19.7"	87°50'03.5"	Net with a telescopic handle	10/2013	No data	4% formalin	ECOSUR, ZL
	18°16'05.9"	87°47'59.5"	Trawl net	01/2007	No data	4% formalin	ECOSUR, ZL
Chetumal Bay-Manatee Sanctuary (RESMBCH)	18°11'27.6"	87°51'36.0	Channel net	07/2024	1	96% ethanol	ECOSUR, ZL
	18°11'01.0"	87°51'26.0"	Net with a telescopic handle	06/1998	No data	96% ethanol	ECOSUR, ZL
	18°11'28.0''	87°51'40.2''	Net with a telescopic handle	01/2016	1	96% ethanol	ECOSUR, ZL

				07/2024			
	18°11'02.6"	87°51'02.5"	Net with a telescopic handle	09/2023	0.85	96% ethanol	ECOSUR, ZL

				03/2025			
	18°11'02.8"	87°51'04.1"	Net with a telescopic handle	09/2023	0.83	96% ethanol	ECOSUR, ZL
	18°12'26.0"	87°51'41.0"	Net with a telescopic handle	10/2023	1	96% ethanol	ECOSUR, ZL
	18°12'35.31"	87°51'2.47"	Channel net	09/2023	1.25	96% ethanol	ECOSUR, ZL
	18°12'28.8"	87°51'41.0"	Net with a telescopic handle	09/2023	0.95-1.5	96% ethanol	ECOSUR, ZL

**Table 2.** Scyphozoa taxa in the Mexican Caribbean. L: literature, CS: citizen science, SC: samples collected. Numbers in parentheses correspond to the reference numbers indicated in the table, columns sting level, ecosystem services and damage incurred. Segura Puertas et al. (1999) (1), Nithiyasri et al. (2025) (2), Kingsford et al. (2017) (3), Muffett et al. (2021) (4), Li et al. (2014) (5), Burnett (1996) (6), Nurasmi et al. (2021) (7), Marambio et al. (2021) (8), Sánchez-Rodríguez and Lucio-Martínez (2011) (9), Segura-Puertas et al. (2002) (10), Morales-Landa et al. (2008) (11), Zare et al. (2023) (12), Brotz et al. (2016) (13), Duarte et al. (2022) (14), Ballesteros et al. (2022) (15), Lucas (2001) (16).

Taxa	ID Worms species	ID BoldSystem	Number of records	Stinging level	Ecosystem services	Damage incurred
*Atolla vanhoeffeni*	135281	without DNA sample	2(L)	no data	no data	no data
*Linuche unguiculata*	221098	without DNA sample	28(L), 13(CS), 1(SC)	moderate (1)	antifungal and antiprotozoal activity (11)	Tourism (1)
*Nausithoe maculata*	287158	without DNA sample	1(L)	moderate (2)	no data	no data
*Nausithoe punctata*	135290	without DNA sample	45(L), 1(SC)	moderate (2)	no data	no data
*Nausithoe rubra*	135291	without DNA sample	2(L)	moderate (2)	no data	no data
*Nausithoe* sp.		without DNA sample	1(CS)	moderate (2)	no data	no data
*Periphylla periphylla*	135294	without DNA sample	1(SC)	low (3)	no data	no data
*Cassiopea andromeda*	135295	without DNA sample	1(L), 1(SC)	low to moderate (4)	anticancer (12), potentially edible resource (13)	Tourism (Aquarists, researchers, recreational snorkellers) (4)
*Cassiopea frondosa*	287166	without DNA sample	6(L), 2(CS)	low to moderate (4)	potentially edible resource (13)	Tourism (Aquarists, researchers, recreational snorkellers) (4)
*Cassiopea xamachana*	287172	MEDUS188-25, MEDUS189-25, MEDUS190-25, MEDUS191-25, MEDUS192-25	27(L), 43(CS), 27(SC)	low to moderate (4)	antiprotozoal activity (11), potentially edible resource (13)	Tourism (Aquarists, researchers, recreational snorkellers) (4)
*Cassiopea* spp.		without DNA sample	69(CS)	low to moderate (4)	potentially edible resource (13)	Tourism (Aquarists, researchers, recreational snorkellers) (4)
*Stomolophus* sp.		without DNA sample	4(CS)	low to moderate (5)	Commercially exploited (fishing or aquaculture) (13, 14)	no data
*Drymonema larsoni*	867502	without DNA sample	6(CS)	very stinging (6)	no data	no data
*Mastigias* sp.		without DNA sample	1(SC)	low (7)	Commercially exploited (aquaculture) (14)	no data
*Chrysaora* sp.		without DNA sample	2(CS)	low to moderate (8)	Commercially exploited (fishing or aquaculture) (13, 14)	Tourism (Aquarists, researchers, recreational snorkellers) (8)
*Pelagia noctiluca*	135305	without DNA sample	7(L), 1(SC)	very stinging (8, 9)	Commercially exploited (aquaculture) (15)	Tourism (Aquarists, researchers, recreational snorkellers) (8)
*Aurelia* spp.		without DNA sample	3(L), 36(CS), 2(SC)	Low (10)	Commercially exploited (fishing or aquaculture) (13, 14)	fisheries, tourism and power stations (16)
*Deepstaria enigmatica*	135307	without DNA sample	1(L)	No data	no data	no data
